# In vivo uptake of antisense oligonucleotide drugs predicted by ab initio quantum mechanical calculations

**DOI:** 10.1038/s41598-021-85453-6

**Published:** 2021-03-18

**Authors:** Henrik Frydenlund Hansen, Nanna Albaek, Bo Rode Hansen, Irene Shim, Henrik Bohr, Troels Koch

**Affiliations:** 1 Roche Innovation Center Copenhagen, Fremtidsvej 3, 2970 Hoersholm, Denmark; 2 Department of Chemistry, B-206-DTU, The Technical University of Denmark, 2800 Lyngby, Denmark; 3 Department of Chemical Engineering, B-229-DTU, The Technical University of Denmark, Lyngby, Denmark

**Keywords:** Biochemistry, Biotechnology, Computational biology and bioinformatics, Structural biology, Molecular medicine, Computational chemistry, Lead optimization, Structure-based drug design

## Abstract

Liver and kidney uptake and antisense activity is studied for a series of Locked Nucleic Acid (LNA) oligonucleotides with fully stereo-defined, internucleoside linkages. These stereo-specific phosphorothioates are made with a newly developed synthesis method and are being analyzed both theoretically and experimentally. Their structures are obtained theoretically by using many-body Schrödinger equations applied to a group of 11 stereo-defined LNA antisense oligonucleotides selected for biological experiments. The fully converged electronic structures were obtained from ab initio quantum calculations providing the specific electronic structures. One important result was the observation that the calculated electronic structure, represented by the *iso-*surface area of the electron density in Å^2^, correlated linearly with LNA oligonucleotide uptake in the liver and kidney. This study also shows that more complex biological phenomena, such as drug activity, will require more molecular and cellular identifiers than used here before a correlation can be found. Establishing biological correlations between quantum mechanical (QM) calculated structures and antisense oligonucleotides is novel, and this method may constitute new tools in drug discovery.

## Introduction

**Figure 1:**
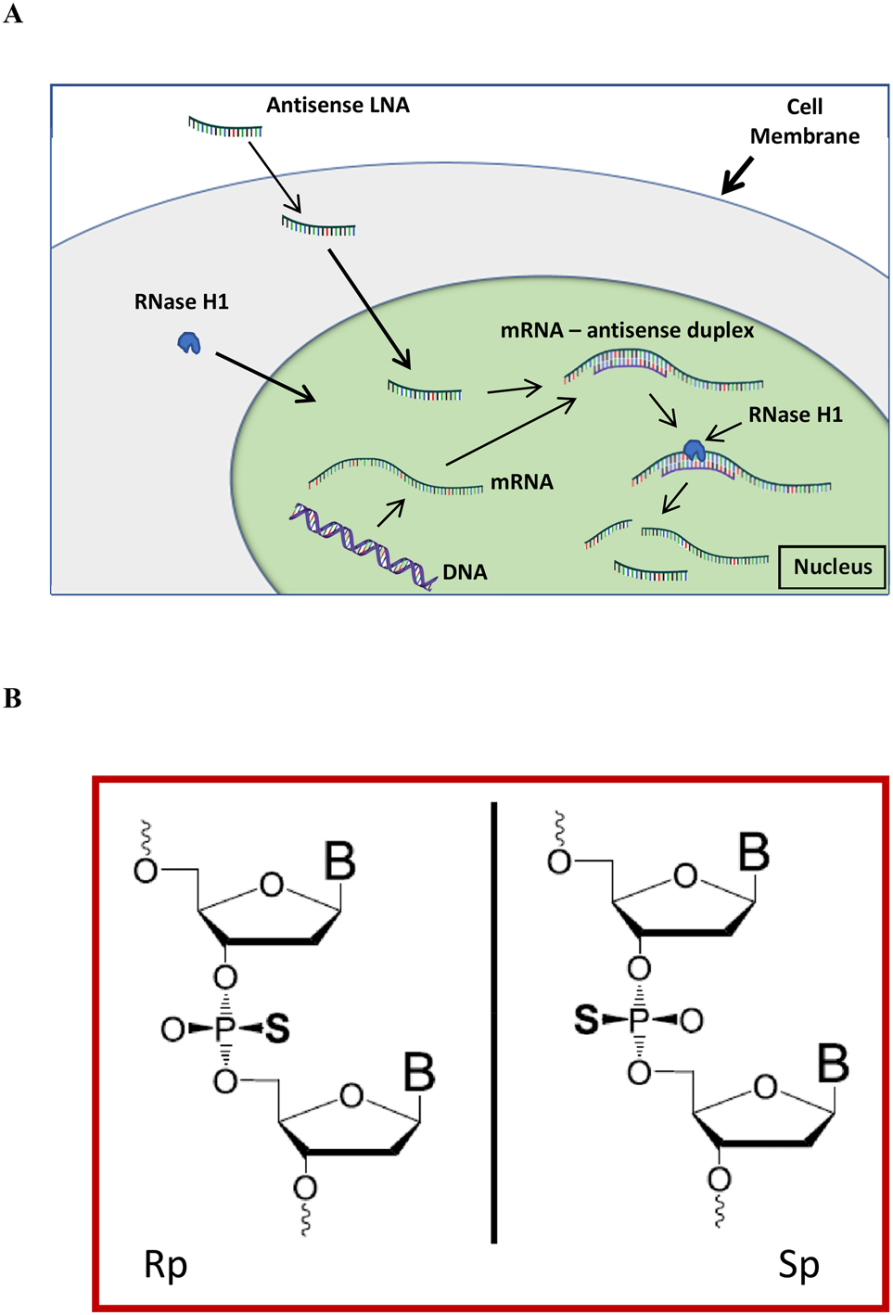
Antisense mechanism (**A**) and phosphorothioate (**B**). (**A**) The use of LNA oligonucleotides as inhibitors of disease—forming proteins, or reduction of harmful RNA, is based on specific hybridization to target mRNA. This duplex recruits the cellular enzyme RNase H1 that degrade the hybridized mRNA. The LNA oligonucleotide remains intact under this process and will repeatedly trigger degradation of further target mRNA; **(B**) the two steric configurations Rp and Sp of the phosphorothioate linkages between the nucleobases of phosphorothioate oligonucleotides.

Specific and potent regulation of RNA has wide perspectives in life sciences and therapeutics^1,2^. In antisense, short single stranded oligonucleotides are designed to target RNA, and by way of design oligonucleotides will hybridize specifically and mediate degradation of the target RNA^3,4^. Degradation of RNA is most efficient in the nucleus where the hybrid duplex between the antisense oligonucleotide and the RNA, will silence RNA by recruitment of the endogenous RNA cleaving enzyme RNase H1 (Fig. 1A). The antisense oligonucleotides used here were Locked Nucleic Acid (LNA)^5,6^. Incorporating LNA nucleosides protects the oligonucleotide better against nucleases, and the high affinity induced by LNA nucleosides translates into higher antisense potency^7^. In most LNA antisense designs the internucleoside phosphates are chemically modified to phosphorothioates (PS)^8^. This backbone modification is crucial since it further enhances nucleolytic stability and improves other drug properties like bioavailability and cellular uptake via increased protein binding by the more lipophilic phosphorothioates^9^. However, with the introduction of each PS internucleoside linkage a chiral center is created at phosphorus (Rp or Sp) (Fig. 1B)^10–12^. Therefore, for every PS linkage introduced in an oligonucleotide, two diastereoisomers are created. Because conventional solid-phase PS synthesis is not stereoselective, an N-mer PS oligonucleotide contains random mixtures of 2^ N-1^ diastereoisomers. In this study a newly developed synthesis method is used for making specific steric configuration of all the phosphorothioate linkages^13–16^. This method reduced the number of diastereoisomers in the tested 13mer LNAs from 2^12^ (4096) to just *one* isomer. Single isomeric PS antisense oligonucleotides (AONs) have major potential in therapeutic use^9,17^. In addition to drug improvements, a transition from "classic" diastereoisomeric mixtures (random mixtures) to single isomeric AONs offers a new opportunity to establish molecule specific correlations between biological—and computational data for PS AONs.

In this work 11 single isomeric LNAs are synthesized from two different nucleobase sequences and designs (Table 1). The LNA oligonucleotides are designed as 13mer gapmers and the original random mixture "parents", were selected for potent antisense activity against the mRNA of respectively ApoB-100 and Hif-1 alpha^7,9,18–21^. From the ApoB-100 random mixture 6 stereo-specific isomers were selected and from the Hif-1 alpha random mixture 5 stereo-specific isomers were selected (Table 1). Naturally, the 11 stereo-specific isomers comprise same nucleoside design, nucleobase sequence, and number of atoms as their respective two "parents"^9, 13,14,17,22^.

**Table 1:** Design, nucleobase sequence, PS chirality and in vivo data for 11 LNAs.

Oligo no	Base sequence	Chiral sequence	Target	Activity 1 mg/kg	Activity 10 mg/kg	Liver uptake 1 mg/kg	Liver uptake 10 mg/kg	Kidney uptake 1 mg/kg	Kidney uptake 10 mg/kg
				% of saline control	% of saline control	LNA µg/g liver tissue	LNA µg/g liver tissue	LNA µg/g kidney tissue	LNA µg/g kidney tissue
LNA 1	G^m^CattggtatT^m^CA	Random mixture	ApoB-100	51.5		0.50		13.2	
LNA 2	G^m^CattggtatT^m^CA	RRSSRSSRSRSS	ApoB-100	19.5		0.85		14.3	
LNA 3	G^m^CattggtatT^m^CA	SRSSRSSRSRSS	ApoB-100	42.4		1.08		16.7	
LNA 4	G^m^CattggtatT^m^CA	RRSSRSSRSSRR	ApoB-100	39.7		0.14		1.90	
LNA 5	G^m^CattggtatT^m^CA	SSSRRRSRRRSS	ApoB-100	86.9		0.78		10.9	
LNA 6	G^m^CattggtatT^m^CA	SRRSRSSRRSRR	ApoB-100	21.0		0.17		3.20	
LNA 7	G^m^CattggtatT^m^CA	RSSRSSRSSRSS	ApoB-100	59.8		1.34		22.6	
LNA 10	G^m^CaagcatcctGT	Mix	Hif-1 alpha	77.9	43.8	0.49	4.89	13.0	68.0
LNA 11	G^m^CaagcatcctGT	RSSRRSRRSRSS	Hif-1 alpha	36.2	18.5	0.48	4.56	13.9	88.0
LNA 12	G^m^CaagcatcctGT	RSRRRSSRSRSS	Hif-1 alpha	77.7	21.8	0.71	4.91	16.1	104
LNA 13	G^m^CaagcatcctGT	RSRRRSSRRSRS	Hif-1 alpha	60.0	61.7	0.57	5.93	13.2	67.5
LNA 14	G^m^CaagcatcctGT	RRSSRSSRSRSS	Hif-1 alpha	51.0	32.7	0.91	6.52	17.0	86.5
LNA 15	G^m^CaagcatcctGT	SRRSRSSRRSRR	Hif-1 alpha	61.4	50.6	0.69	7.00	18.5	103

LNA and DNA nucleotides are respectively denoted by upper and lower case letters. For LNA nucleotides all cytosine nucleobases are replaced by 5-methyl-cytosine (^m^C). LNA nucleobase/PS chiral sequence and target information is shown in the columns to the left; antisense activity is shown at 1 and 10 mg/kg in the middle of the table; LNA uptake in liver and kidney at 1 and 10 mg/kg is shown in the 4 columns to the right.

We have previously investigated smaller sized 8mer PS LNAs with respect to their electronic structures^23, 24^. With these "model" sized LNAs it was demonstrated that optimized, quantum mechanical (QM) ab initio calculations, provided an overall correctness in the structure and electrostatics. These calculations also illustrated that single PS R or S chirality "mutations" induced profound structural and electrostatic changes.

QM modelling of biological relevant AONs has not hereto been made, and there has never been established a correlation between purely structure-based biophysics and pharmacological properties of AONs. Obtaining fully converged ab initio quantum mechanical (QM) calculated structures of such large molecules is an accomplishment that provides new information about oligonucleotide molecules (Table 2).

**Table 2:** Calculated biophysical parameters of the 11 stereo-defined LNAs.

Oligo no.	Dipole moment (D)	Distance 5′O-3′O (Å)	Electron density area (Å^2^)	Potential surface area (pos) (Å^2^)	Potential surface area (neg) (Å^2^)	Potential surface area exposed (pos) (Å^2^)	Potential surface area exposed (neg) (Å^2^)	HOMO (eV)	LUMO (eV)
**LNA 1**									
LNA 2	47.5	39.9	3229	3884	1726	1516	287	− 8.14	0.24
LNA 3	43.4	22.8	3253	3981	1838	1447	246	− 8.23	0.18
LNA 4	29.5	17.8	3176	3686	1682	1370	124	− 7.93	− 0.41
LNA 5	33.2	26.9	3194	3703	1975	1442	192	− 8.00	− 0.14
LNA 6	43.6	20.7	3231	3738	1745	1443	139	− 7.91	− 0.39
LNA 7	74.6	46.7	3322	3946	1900	1684	313	− 8.11	− 0.72
**LNA 10**									
LNA 11	48.0	27.1	3100	3709	1811	1357	262	− 7.71	0.42
LNA 12	8.7	30.8	3153	3576	1715	1396	181	− 7.97	− 0,13
LNA 13	23.3	27.6	3127	3568	1740	1320	194	− 8.02	0.05
LNA 14	23.9	17.2	3205	3859	1788	1477	267	− 8.11	0.30
LNA 15	49.1	21.7	3102	3681	1983	1439	284	− 7.85	− 0.63

The columns from left to right shows the LNA numbering, the Dipole moment in Debye (D), the distance measured from the 5′-O (in the 5′-OH) to 3′-O (in the 3′-OH), the area measured on the surface of the electron density of *iso-* value 0.002 electrons/a.u^3^. Next two columns show the area of the positive and the negative electrostatic potential surfaces of numerical value 83.68 kJ/mol. Similar results are shown for the exposed potential surfaces. Last two columns contain the energies of HOMO and LUMO.

## Results

### Biophysical data

**Figure 2:**
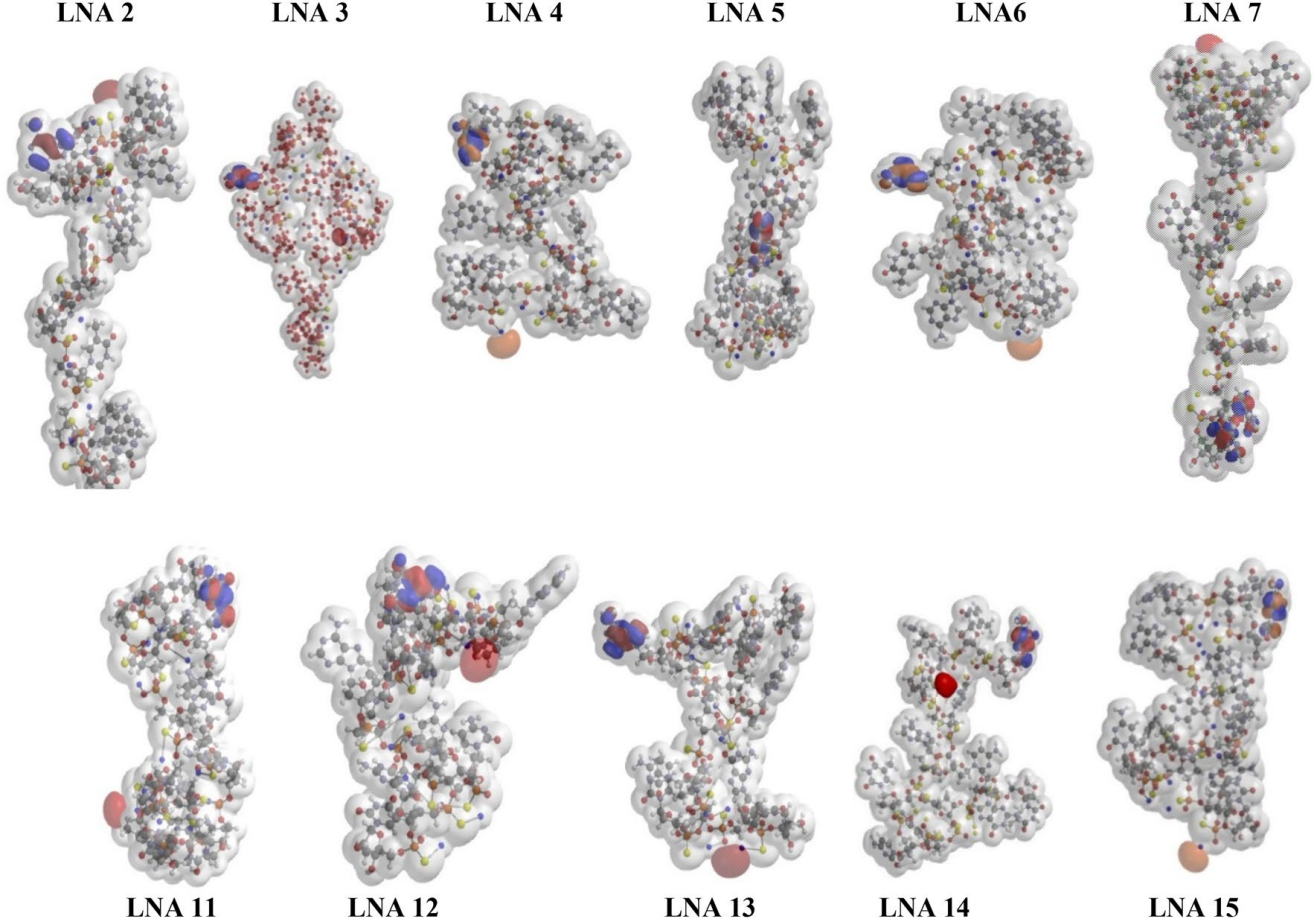
Top row shows the optimized structures of LNA oligonucleotides targeting ApoB-100, lower row shows the corresponding results for the LNA oligonucleotides targeting Hif 1-alpha. The figures include the electron density *iso*-surface at 0.002 electrons/a.u.^3^ illustrated by the partly transparent surface. The Frontier orbitals (HOMO and LUMO) for each LNA oligonucleotide are shown as spheres respectively in blue-red and orange-red.

Specificity and correctness of the results from HF-SCF (Hartree–Fock self-consistent field) optimizations has previously been demonstrated (see Fig. 2 in Ref.^24^)^23–26^. Each structure is unique and starting from different initial values identical converged structures were produced, with a difference in electron densities of less than 1/1000^24^. The 11 stereo-defined LNAs exhibited great diversity in the final converged structures (Table 2 and Fig. 2), demonstrating sequence dependence and that structure and biophysical observables are highly specific for a given PS "chiral sequence" (Table 1). It was found in all data set that a single PS R or S chiral "mutation" could significantly impact the properties. For instance, a single PS R or S chirality mutation changed the 5′-O-3′-O distance 17,1 Å (Table 2, LNA 2 and 3), and the structures of LNA 2 and 3 are also clearly, visibly, different (Fig. 2). It was observed that molecules with calculated same lengths could have very different antisense activity (Fig. S2 and Table 1, LNA 11 and 13), and that molecules with the same activity could have different length (LNA 13 and 15). The length of the ApoB-100 compounds was on average 30% longer than the Hif-1 alpha compounds.

The Dipole moment (Table 2) is derived from atomic charge distributions in the wave function, and it describes the average charge separation. LNA 12 had the lowest dipole moment (8.7 D) and LNA 7 the largest (74.6 D). There seems no clear correlation between the dipole moment and the biological properties.

The positive and negative electrostatic potential surface areas were calculated (*iso*-value = 0.002 electrons/a.u^3^) (Table 2). The potentials fix an energy for the field that a test charge is exposed to at a certain three-dimensional distance around the molecule and give a measure of the energies involved at the surface of the molecule. The electrostatic potential surface areas were calculated either for all atoms or for the ones exposed (Spartan user guide p 245). The positive area (*i.e.,* electron poor region) is almost twice as large as the negative area (*i.e.,* the electron rich region), but that does not apply to the exposed areas where the positive area was approx. 5 times larger than the negative areas.

The spatial (geometrical) position of the highest occupied molecular orbital (HOMO) and the lowest unoccupied molecular orbital (LUMO) are mostly at opposite ends of the molecules (Fig. 2). This indicates that electrons are more delocalized, or mobile, and positioning at either end is giving rise to large dipole moments. This is especially observed for the longest molecule LNA 7 exhibiting by far the largest dipole moment but also seen for LNA 11 and 15. The HOMO energies vary only approx. 5% (∆ eV = 0.43 eV). The LUMO energies are naturally higher and vary a bit more (∆ eV = 1.05 eV) (Fig. 2).

### Target down-regulation in vivo

Target RNA knockdown for all the tested compounds in mice liver are listed in Table 1 and shown in Fig. 4 and Fig. S2. Miniature representations of the energy minimized, and fully converged structures are included in the figures. Mice were dosed once intravenously (*i.e.*) with 1 or 10 mg/kg for Hif-1alpha—and 1 mg/kg for the ApoB-100 compounds. For both targets antisense activity varied significantly. ApoB-100 mRNA levels in liver varied between 19.5 and 86.9%—compared with saline control (100%). The most potent compound (LNA 2) was a factor of 2.6 more effective than the parent random mixture (LNA 1), whereas LNA 5 was a factor of 1.7 less active. A single PS R or S chirality mutation influenced activity a factor of 2.2 (Fig. 4 and Table 1; LNA 2 and LNA 3).

Liver down regulation of Hif-1 alpha mRNA followed the same pattern and varied from 36.2 to 77.7%—compared with saline control (100%). A difference of a factor of 2.1 was observed between the most and the least active compounds (LNA 11 and 12 + the random mixture; Table 1 and Fig. S1). In general, Hif-1-alpha was not as sensitive for antisense down regulation as ApoB-100. A high dose of 10 mg/kg was necessary to reach the same range of knockdown.

### Correlation between the electron density area and LNA accumulation in liver

**Figure 3:**
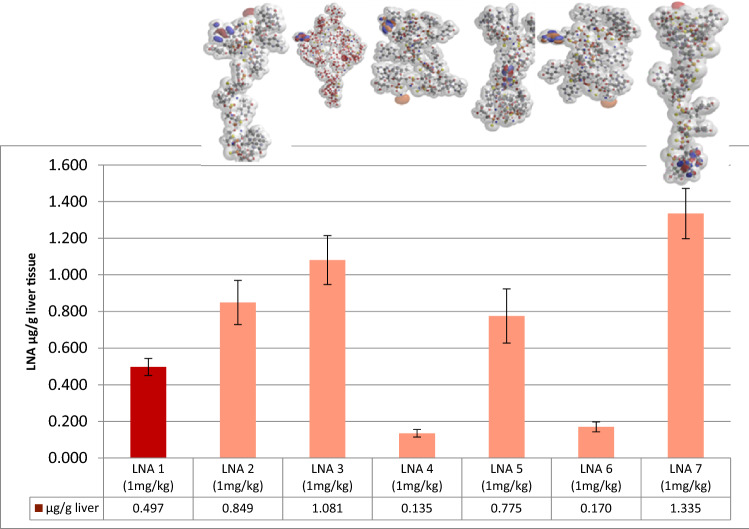
Liver uptake of LNA diastereoisomers targeting ApoB-100. Diversity of diastereoisomers on oligonucleotide concentration in liver of mice (C57BL/6qBom) (n = 5) dosed once intravenously at 1 mg/kg at day 0 and taken down day 3 (60 h). LNA oligonucleotide concentration (µg/g tissue) determined by hybridization-ELISA. Dark red color LNA 1 shows random mixture of stereoisomers. Light color show stereo-defined isomers with identical nucleobase sequence and nucleoside design as LNA 1.

**Figure 4:**
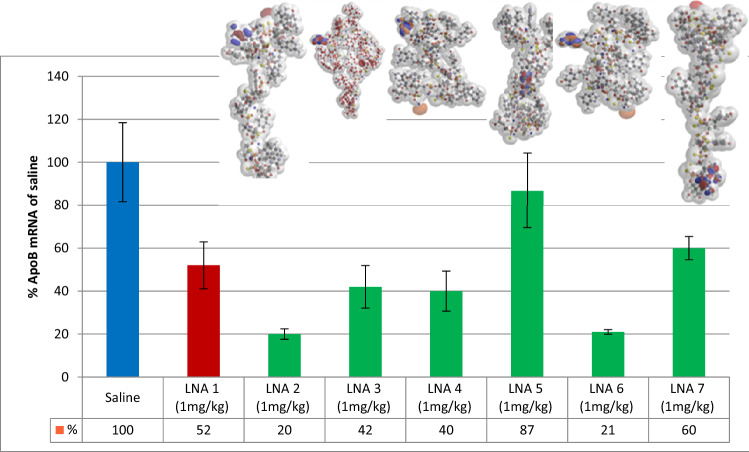
Antisense activity of LNA diastereoisomers targeting ApoB-100. Diversity of diastereoisomer knockdown of target ApoB in livers of mice (C57BL/6qBom) (n = 5) dosed once intravenously at 1 mg/kg on day 0 and taken down day 3 (60 h). Target mRNA level (% of saline control) were measured in liver tissue. Blue, saline control; red LNA 1 random mixture of stereoisomers. Green, stereo-defined isomers with identical nucleobase sequence and nucleoside design as LNA 1.

Substantial variations in liver content were found. At the same dose no direct correlation between liver content and the target knockdown was found *among* the tested compounds. Thus, molecules with low uptake can have high antisense activity (LNA 6 and LNA 11, Figs. 3, 4 and Figs. S1, S2).

**Figure 5:**
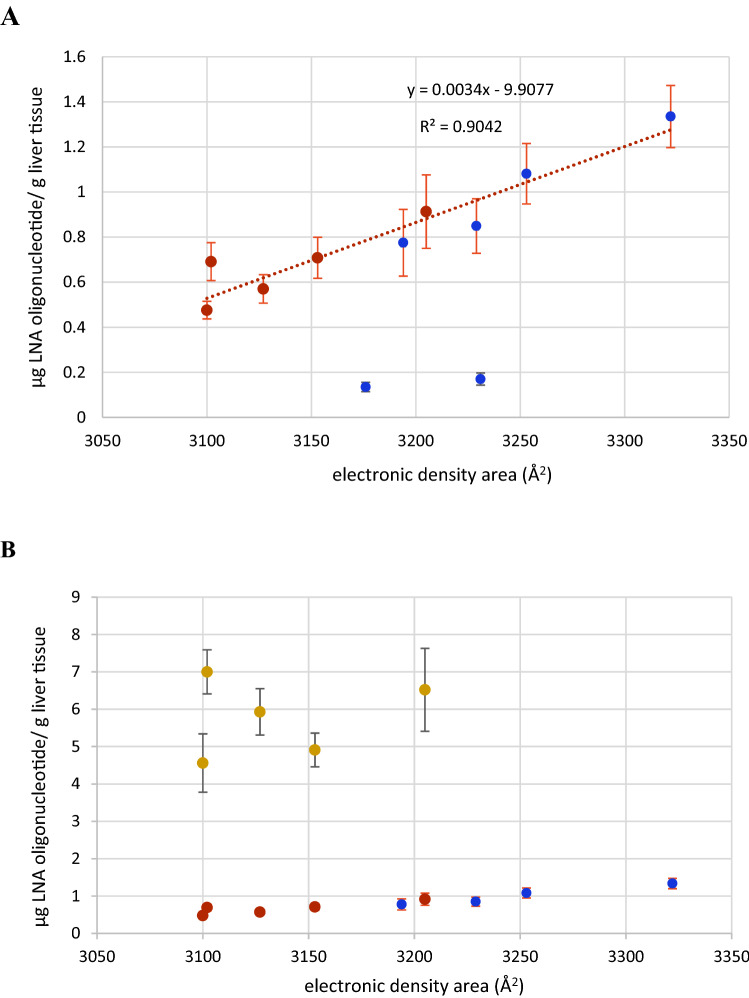
Correlation between electronic density area and liver uptake in mice (C57BL/6qBom) for all 11 stereo-defined LNAs. Six stereo-defined LNAs against ApoB-100 (blue circles) and five stereo-defined LNAs against Hif-1alpha (green circles). (**A**) Red: Hif-1 alpha. Blue: ApoB-100, all LNAs dose at 1 mg/kg; (**B**) All ApoB-100 LNAs dosed at 1 mg/kg (blue); all Hif-1 alpha LNAs dosed at 1 mg/kg(red) and 10 mg/kg (yellow). The R^2^ values includes measurements of n = 5 mice for liver content for all LNA oligonucleotides. LNA 4 and 6 are not included in the statistics since the measured amount does not represent truly the uptake due to fast 3′ degradation.

In contrast to antisense activity, we were able to establish a correlation between the QM calculated parameter *electron density area*^27,28^ (Table 2) and LNA uptake (Figs. 3, 5A; Figs. S1, S5 and S6). Liver uptake correlated linearly with the electron density area in Å^2^ for 9 of the tested LNAs at the 1 mg/kg dose (Fig. 5A). However, 2 of the isomers (LNA 4 and LNA 6) were found not to correlate. When the molecular designs and steric properties of these two compounds were examined it appeared that both had PS configurations RR at the 3′-ends and a terminal LNA nucleoside adenosine. These structural components are known to be among the most labile compositions against *exo*-nucleolytic degradation^29,30^. To test this, all LNA oligonucleotides were incubated in rat serum for 4 h (Table S1). Except for the non-LNA oligonucleotide PS AON control, LNA 4 and LNA 6 were by far the most labile and exhibited a similar degradation of respectively 19.6% and 19.2%. The main degradation product was established to be n-1 (Figure S3). All the other isomers were virtually nuclease resistant in rat serum (Figures S3 and S4). It has been demonstrated in rat hepatocytes that oligonucleotides, incl. LNAs, follow a degradation pattern closely related to the pattern found in *exo*-nuclease assays^30^. We therefore conclude that the much lower accumulation of LNA 4 and LNA 6 at the point of detection at day 3 (Fig. 3 and Table S1) is due to degradation of the full-length compounds (Fig. S3). We conclude this since the hybridization ELISA methodology used here for tissue content quantification is sensitive for full length detection and thus, n-1 deletion products are virtually not detected (Fig. S3)^7^. However, we were not able to correlate uptake in liver to the electron density area at the high dose (10 mg/kg) for the Hif-1alpha compounds (Fig. 5B). At this dose antisense activities for the Hif-1alpha compounds did not increase proportionally with dose, even though accumulation did. Therefore, the Hif-1 alpha LNAs have activity/pharmacology-wise reached saturation levels in liver^7^.

### Correlation between the electron density area and LNA accumulation in kidney

**Figure 6:**
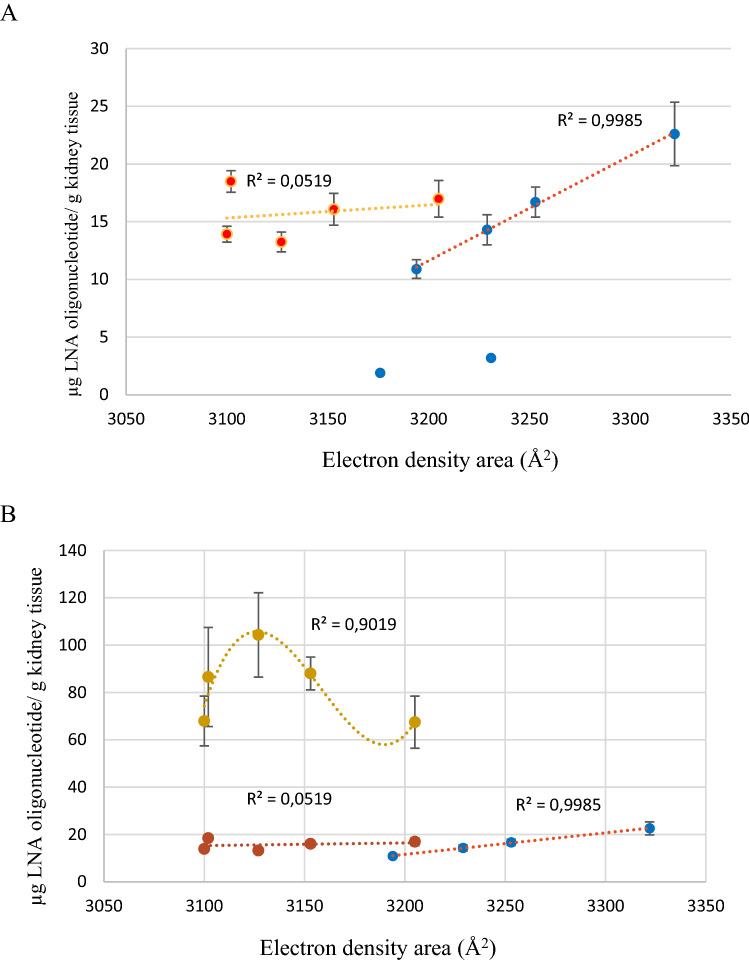
Correlation between electronic density area and kidney uptake in mice (C57BL/6qBom) for all 11 stereo-defined LNAs. Six stereo-defined LNAs against ApoB-100 (blue circles) and five stereo-defined LNAs against Hif-1alpha (green circles). (**A**) Red: Hif-1 alpha. Blue: ApoB-100, all LNAs dosed at 1 mg/kg; (**B**) All ApoB-100 LNAs dosed at 1 mg/kg (blue); all Hif-1 alpha LNAs dosed at 1 mg/kg (red) and 10 mg/kg (yellow). The R^2^ values includes measurements of n = 5 mice for liver content for all LNA oligonucleotides. LNA 4 and 6 are not included in the statistics since the measured amount does not represent truly the uptake due to fast 3′ degradation.

When we correlated the electron density area and uptake in kidney a different picture emerged compared to liver (Fig. 6). At the 1 mg/kg dose four ApoB-100 LNAs correlated linearly with high significance (R^2^ = 0.99) (Fig. 6A and Fig. S5). LNA 4 and 6 were detected at day 3 in much lower concentrations due to nuclease degradation (vide supra). The two sets of isomers accumulated in the same range, 10–20 μg/g, but in contrast to the ApoB-100 compounds, we were not able to establish a correlation between the electron density area the tissue accumulation of the Hif-1 alpha compounds. Generally, the kidney accumulated much more oligonucleotide than the liver. The increased accumulation varied from 14 to 26 times more oligonucleotide for the ApoB-100 compounds, and it varied between 22 and 26 times more for the Hif-1alpha compounds. For the Hif-1alpha compounds at the high dose, 10 mg/kg, the 10 times higher dose accumulated 5–6 times more LNA. Curve fitting produced a higher order correlation with a R^2^ = 0.90 between kidney accumulation and the electron density area.

## Discussion

### Rationale and Justification of the applied QM methodology

Computational QM techniques provide the possibility to understand electronic structures in detail. The wave-function describes the full electronic solution of physical information about the electronic system under the given Born–Oppenheimer approximation. Quantum mechanics teaches us that the wave function contains all the information that can be known about the system, and thus, there exist a unique mapping between the electron density and all properties of the system^28^. This gives a strong theoretical justification for choosing electron density as parameter for studying quantitative structure–activity relationship or quantitative structure-properties relationship (QSAR/QSPR)^28^.

However, the essential question for using the applied QM methodology in biological correlations is to verify the validity of the calculated biophysical parameters, and to address the issue, to what level the obtained molecular structures are true representatives and relevant for a biological system. We have previously accounted for the "correctness" using this QM methodology^24^, but in addition to this, two other questions are important to address in order to ascertain correctness: 1, How will the selected *iso*-value influence the obtained structures; 2, How will different conformations of the individual molecules influence the calculated biophysical properties and structures.

1. The QM calculations were performed using an *iso*-value of 0.002 electron/au^3^. For the construction of the *electron density area* this value is not crucial; in contrast to the *iso*-value(s) chosen for defining the *electrostatic potential surface*^27^*.* The former value is often chosen to be 0.002 electron/au^3^, but other values are not changing the electron density area much. For instance, via a doubling of the *iso*-value is changing the electron density area with less than 1%, underlining the consistency of the data.
2. Oligonucleotides can attain different conformations other than the illustrated fully convergent structures. Compared to proteins, optimization of single stranded DNA and LNA quantum calculated structures is less complicated as they are more rigid structures, and their conformational sub-states are more even in energy^24^. The calculations of the ground states of DNA and LNA turned out to be quite unique since their conformational states are within or less than 1 eV (= 0.037 au; where 1 au = 27.21 eV). This is less than the difference in energy between the chiral isomers of one of the isomer set (ApoB-100 or Hif-1alpha) of around 1.63 eV (= 0.06 au), and much less than the energy difference between the isomers of different isomer sets with different sequence which is about 1632 eV (= 60 au). Therefore, changing between different conformational states will not significantly change the electron density area.

The electron density area is an important determinant for the electronic properties of the outer surface of a molecule (vide supra). A larger area comprises more delocalized electrons and will interact more with the surroundings^31–33^. When the size of the electron density area is compared with the compound structures (Fig. 2) it appears that a larger area is linked with exposed nucleobases. Exposed nucleobases offer a larger possibility for protein binding, via e.g., H-bonding, van der Waals forces and π-π-stacking. Hence, the probability for protein binding increases for a given molecule when the electron density area becomes larger^28,33^. Oligonucleotide binding to membrane proteins is a driver behind cellular uptake and therefore, the electron density area is important for uptake.

### Electron density area: correlation with tissue accumulation and uptake mechanisms

Pharmacokinetics and cellular uptake of PS oligonucleotides, including LNAs, is well established^34–39^. Due to the lipophilic nature of the PS backbone PS AONs bind extensively to plasma proteins, e.g., serum albumin and γ-globulin^36^. The binding constants are relatively low (K_d_ in micromolar range), but it is large enough for protein binding that enables little renal clearance and facilitating efficient uptake in many tissues^35–37^. Following either *i.v.* or *s.c.* administrations plasma concentrations decline rapidly in a few hours in a multi exponential manner, followed by a much slower tissue elimination phase^35, 39^. The tissue half-lives are species dependent and long—typically 2–8 weeks^9, 34–36, 39^.

AONs cross readily the endothelium in most tissues by diffusion through intercellular routes or by transcytosis across the endothelium^35, 37^. The uptake in the cells (hepatocytes) of the fenestrated endothelium in livers is particularly efficient. When AONs reach cells (e.g., hepatocytes) they will associate with and bind to membrane proteins and get internalized predominantly via endocytosis^37,38,40–42^. This is a complex process where several pathways and many membrane proteins are engaged^37,38, 40,41,43–46^. It has been shown, dependent on the plasma concentrations presented to the liver that productive as well as nonproductive uptake mechanisms exist in the cells^37,38,40^. This is one of the reasons for that antisense activity does not necessarily correlate linearly with bulk uptake^9, 37,38^.

In this study all administrations are given as a single *i.v.* dose, and at each dose level the initial plasma concentrations are similar for all compounds. We measure the final tissue concentration at day 3, which is a long time after the plasma half-life (a few hrs.), but long time before the tissue elimination half-life (2–8 weeks). We also know that except for LNA 4 and 6 the compounds are negligibly degraded at day 3. Therefore, at day 3 LNA tissue accumulation is representative for uptake profile and mechanism.

We see pronounced differences in antisense activity and uptake in both liver and kidney (Table 1). This is in accordance with many other observations (vide supra), and high uptake did not correlate with high antisense activity (Fig. 2 and Table 1). It is important to note that this significant property diversity among the structurally similar compounds was caused by few "mutations" in the PS chirality of the backbone. Thus, a few chirality modifications can influence and improve important pharmaceutical properties illustrating the prospects of using stereo-defined PS AONs in drug discovery^9, 17^.

We were able to establish a linear correlation at the 1 mg/kg dose between the electron density area and liver uptake. This was seen for both the ApoB-100 and Hif-1alpha compounds. Thus, in liver, at this dose, the electron density area appears to predict drug accumulation. The linearity could indicate that the LNAs are taken up by the same molecular mechanism. At the high dose, 10 mg/kg, a lack of linearity was observed for the Hif-1alpha compounds. Since the high dose produced approx. 10 times more drug accumulation, uptake is not influenced by saturation. Apparently at this dose, the plasma concentration limit has been exceeded for which the uptake mechanism related with the electron density area is simple (linear), and it has become more complex, e.g., by activating multiple uptake mechanisms (vide supra).

When we consider the electron density area and uptake in kidney at the 1 mg/kg dose, we were able to establish a linear relation for the ApoB-100 compounds, but not for the Hif-1alpha compounds (Fig. 6). For the Hif-1alpha compounds we were not able to establish a linear relation at either 1 mg/kg or at 10 mg/k. Interestingly, the ApoB-100 compounds exhibited a linear correlation between the electron density area and uptake with very high significance (R^2^ = 0.99). The linear correlation could indicate that ApoB-100 compounds were taken up by the same mechanism. Oligonucleotides are predominantly taken up in kidney by the cortex, and apparently, the ApoB-100 compounds interact differently with kidney cells than the Hif-1alpha compounds do. Kidney and liver exhibit different uptake mechanisms and dynamics, in part confirmed by the fact that the kidney accumulates approx. 15 times more than liver (Table 1). The consequence here is that one sequence set of isomers (ApoB-100) exhibited a different uptake profile/mechanism than that of the other isomer set (Hif-1alpha)—at the same initial plasma concentrations. However, there might be a relation of higher order for the Hif-1alpha LNAs at the 10 mg/kg dose. In any case, the uptake profile exhibited high sensitivity for changes in the electron density area.

Concerning the activity of the compounds it is interesting that the two LNAs that are among the most active (LNA 4 and LNA 6) also are the most degradable. The fact that LNA 4 and LNA 6 was detected with the least amount accumulated in the liver at day three is due to degradation by 3′*exo* nucleases of the full length 13mer. Despite the low content measured at day three the oligonucleotides were probably taken up in comparable amounts with the other LNAs at the beginning of the experiment. We know that LNAs are taken up in large numbers by cells and in large excess to the RNA targets in the nucleus^42,47^. Therefore, even if a big portion of cellular LNA is degraded there will still remain oligonucleotide present to antagonize the target. Most importantly, we have shown that the two 13mer ApoB-100 targeting LNAs (LNA 4 and LNA 6) are degraded by 3′ *exo* nucleases producing the n-1 12mer truncations. We have earlier shown for the corresponding random mixtures that the 12mer is just as active as the 13mer^7^. Therefore, it is not surprising also to get activity for these two stereo-specific 12mer isomers.

Our data showed that the overall structure of LNA oligonucleotides is strongly dependent on the composition and chirality of the PS backbone. Changing just one chiral PS position has dramatic effect on both antisense activity and uptake, but it does not result in a *specific* change leading to a unique structure of the single strand. It has previously been suggested that LNA nucleotides pre-organize both single strands and LNA hybrid duplexes^48–50^. These studies are based on NMR and thermal denaturation research illustrating that favorable entropy contribution is a major driver for the LNA high affinity. However, in these studies little has been shown how this pre-organization influences the overall *specific* structure of the single strand. Data from our earlier work also show that inclusion of LNA nucleotides does not lead to a *specific* unique structure of the single strand^24^. Thus, we see no similarities in length and helicity that might have been anticipated for LNA gapmers.

In summary, the electron density area may be used to predict uptake accumulation and to differentiate uptake mechanisms of AON stereoisomers. We showed here a sequence independent accumulation prediction in liver, and that sequence independent linearity exists within a certain range of dose/plasma concentration. In the kidney that accumulated approx. 15 times more than liver a different profile was found. Here, linearity was also found, but it was sequence dependent. Further studies will have to be conducted to establish the range of the dose and plasma concentrations producing uptake linearity, and how this is reflected in the different tissues.

These relations were shown for diastereoisomers with the same length. Studies in a broader structural AON space (sequence length and design) and dose/concentration range will have to be conducted in order to get a better understanding of the structural generality, and finally, the predicting power of the electron density area.

Today, in silico bioinformatics is intensively used in RNA therapeutics. Bioinformatics are based on a "four letters" nucleobase representation of AONs (A, T/(U), G, C), and it is remarkable how much targeting information and property prediction that has been extracted from these algorithms regarding classic random diastereoisomeric mixtures. However, more molecular specific information is needed to fully understand protein/membrane binding, intracellular trafficking, and uptake. The QM calculation methodology shown here could be a first step in that direction. It is based on an exact molecular assignment and extracts the biophysical information from the atomic and electronic levels—the fundamentals of molecules. In the future, where computer power will be greatly expanded, QM methodology provides the potential to guide drug discovery and property prediction to a new level. That may turn out to be an important step forward for drug discovery.

## Conclusion

Quantum Mechanics (QM) calculations and electronic structure elucidation is demonstrated for relatively large, biologically relevant, phosphorothioate, antisense oligonucleotides. In vivo experiments showed that the tissue uptake in liver and kidney can be correlated with the area of electron density obtained from the calculations. This was demonstrated for 13mer stereo-defined LNA oligonucleotides comprising two different nucleobase sequences targeting two different RNA targets. QM methodology like this has not been applied to antisense oligonucleotides before. The generated data are based on exact molecular assignments and are unique. This may turn out to be a new way to obtain in silico molecular specific information relevant for biological systems.

## Material and methods

### Oligonucleotide synthesis

The synthesis of chiral DNA and LNA 3′-O-oxazaphospholidine monomers was performed using previously described methods^13–15^. The oligonucleotides (13mers) of common sequence were either 5′-G^m^CattggtatT^m^CA-3′ (ApoB-100) or 5′-G^m^CaagcatcctG^m^T-3′ (Hif-1-α), and the Rp and Sp *iso*forms were synthesized according to published procedures with the exception that DCI (4,5-dicyano imidazole) was used as activator. Anion exchange (AIE) chromatography was performed on an Dionex Ultimade 3000 system. Column: DNA-pac™ PA100, 2 × 250 mm. Solvents: Buffer A (10 mM NaClO4, 1 mM EDTA, 20 mM TRIS–HCL pH 7.8). B (1 mM NaClO_4_, 1 mM EDTA, 20 mM TRIS–HCL pH 7.8). Gradient 0 min. 0% B, 35 min. 35% B, 40 min. 0% B. Detection 260 nm. 50 µL injected.

The purity and mases of the oligomer products analyses were performed on a Waters Acquity UPLC BEH C18 column (1.7 µm, 2.1 × 150 mm) using Buffer A (2.5% methanol in 0.2 M hexafluoro isopropanol, 16.3 M triethyl amine in water) and Buffer B (60% methanol in 0.2 M hexafluoro *iso*-propanol, 16.3 M triethyl amine in water) at a flow rate of 0.5 mL/min with the following gradient program: 10% B for 0.5 min, 10–30% B in 4.5 min, 30% B in 1 min, 30–100% B in 1 min, 100–10% in 1 min. Temperature 65 °C. Injected sample 2 µL. Components were detected based on absorption at 260 nm. Areas of full-length and degradation products were integrated, and longer chain fragments were interpreted based on electro spray mass spectrometry analysis. The areas of the degradation products were adjusted with respect to their nucleobase composition^47^.

This UPLC LCMS method separate n-1 and n + 1 products very well from main sequence (Figs. S3, S4). The full-length products were > 98%. The molecular weight of LNA 1 and its diastereomer versions LNA 2–7 were found to be 4325. The mases of LNA 10 and its diastereomer versions LNA 11–15 were found to be 4253.3. Both in agreement with theoretical/calculated values. The thermal denaturation temperature was measured for the two "parent" random mixtures LNA 1: 57 °C and LNA 10: 62 °C against the corresponding RNA complement^7,51^.

### Oligonucleotide stability

LNA oligonucleotides were added to rat serum to a target concentration of 22.9 µM. Samples were incubated for 4 h at 37 °C in UPLC vials. The mixture was then analyzed by reversed phase Ultra Performance Liquid Chromatography coupled with Mass Spectrometry (UPLC-MS). Analyses were performed on a Waters Acquity UPLC BEH C18 column (1.7 µm, 2.1 × 150 mm) using Buffer A (2.5% methanol in 0.2 M hexafluoro isopropanol, 16.3 M triethyl amine in water) and Buffer B (60% methanol in 0.2 M hexafluoro *iso*-propanol, 16.3 M triethyl amine in water) at a flow rate of 0.5 mL/min with the following gradient program: 10% B for 0.5 min, 10 – 30% B in 4.5 min, 30% B in 1 min, 30–100% B in 1 min, 100–10% in 1 min. Temperature 65 °C. Injected sample 2 µL. Components were detected based on absorption at 260 nm. Areas of full-length and degradation products were integrated, and longer chain fragments were interpreted based on electro spray mass spectrometry analysis. The areas of the degradation products were adjusted with respect to their nucleobase composition^47^. Only LNA n-1 degradation products were found and identified (Figs. S3 and S4).

### In vivo experiments

In vivo experiments were conducted according to the European standards and protocols were approved by the Danish National Committee for Ethics in Animal Experiments.

We performed experiments on 2 classes of samples including the sequences with mixed chirality and 6 or 5 oligonucleotides respectively of the same sequence but different chirality.

The ApoB-100 experiment: Five female—C57BL/6qBom mice in each group were given one iv dose of 1 mg/kg. After 60 h the mice were sacrificed, and liver samples were analyzed by qPCR for determination of ApoB-100 mRNA according to procedure described in and LNA oligonucleotide content according to procedure described in references^7,9^. The Hif-1-α experiment: Five female—C57BL/6qBom mice in each group were given one dose iv of either 1 mg/kg or 10 mg/kg. After 60 h the mice were sacrificed, and liver samples were analyzed by qPCR for determination of ApoB-100 mRNA according to procedure described in reference^7^ and LNA oligonucleotide content according to a hybridization ELISA procedure described in reference^7^.

### Computational techniques

The electronic solution to the Hamiltonian energy equation for a large system of atoms in a complexed bio-molecule can be expanded in a fixed set of basis functions. It consists of a variable superposition of all determinants of the N-particle combinations from the Fock space considered as the full configurational interaction space. The wave-function describing the full electronic solution will in principle contain all the available physical and chemical information of the electronic system and under the given approximation of the Born–Oppenheimer with the particles separated in slowly moving nuclei and with fast electrons moving freely in an average field of the other particles. In the self-consistent field method the missing correlation energy is adjusted for by the SCF-HF scheme, presently used. The wave-function and the energy spectrum are derived from the static time-independent Schroedinger equation together with the Roothaan-Hall equations. In Quantum Monte-Carlo method (QMC) a temperature like parameter can be adapted making the standard static method appear as a zero temperature method.

The technical details for carrying out these large quantum chemistry calculations is described in references 25–27—the latter being program descriptions. It is unusual that such relatively large molecules with hundreds of atoms and several thousand electrons in many energy states can be calculated using full scaled QM techniques. The LNA oligonucleotides targeting ApoB-100 and Hif-1 alpha comprise respectively 442/433 atoms and 2358/2320 electrons. The wave-functions and the electronic structures of the 13mers are derived using the *ab-initio* techniques^27^ of the Hartree Fock, self-consistent field method, HF-SCF, in the SPARTAN /GAUSSIAN program packages. The method is based on solving the time independent electronic Schrödinger equation. The particular oligonucleotides were constructed by using the nucleotide builder in Spartan^27^. The start configuration is a single stranded helix with a rise of 2.55 Å and rotation per base of 32.7 degrees. This start configuration with the particular structural modification such as chirality of the phosphorothioates for the specific LNA oligonucleotides with addition of sodium is then being optimized using the HF-SCF method to get a resulting wave-function describing the electronic orbitals of the molecule. For the calculation of the electron density area, we have used an *iso*-value of 0.002 electrons/(a.u.) for establishing the *iso*-surface.

### Statistical analyses

Standard deviations is based on the entire population and calculated from the STDEV.P in Excel. Linear regression analysis was done using Excel linear regression.

## Supplementary Information

Supplementary Information
