# Supplementary material for: In vivo uptake of antisense oligonucleotide drugs predicted by ab initio quantum mechanical calculations
Source: Sci Rep. 2021 Mar 18;11:6321. doi: 10.1038/s41598-021-85453-6 (PMC7973520; doi:10.1038/s41598-021-85453-6)
Supplement: Supplementary file 1 — Supplementary Information [file 41598_2021_85453_MOESM1_ESM.pdf]

SUPPLEMENTARY MATERIAL

In vivo uptake of antisense oligonucleotide drugs predicted by *ab initio* quantum mechanical calculations

Henrik Frydenlund Hansen[a], Nanna Albaek[a], Bo Rode Hansen[a], Irene Shim[b], Henrik Bohr[c*] and Troels Koch[a]

[a]Roche Innovation Center Copenhagen, Fremtidsvej 3, DK-2970 Hoersholm, Denmark
[b]Department of Chemistry, B-206-DTU, The Technical University of Denmark, DK-2800, Lyngby, Denmark
[c]Department of Chemical Engineering, B-229-DTU, The Technical University of Denmark
*Correspondence should be addressed to: hbohr@dtu.dk

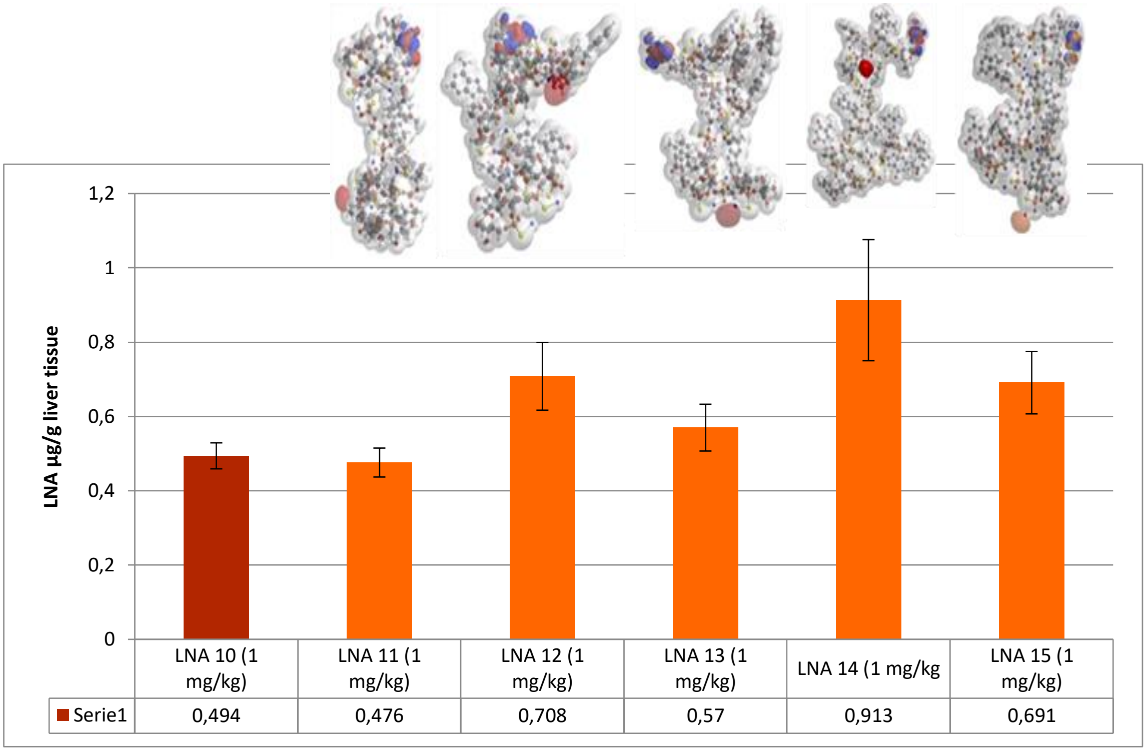

Figure S1. Liver uptake of LNA diastereoisomers targeting Hif-1alpha. Diversity of diastereoisomer effect on oligonucleotide concentration in liver of mice (C57BL/6qBom) (n=5) dosed once intravenously at 1 mg/kg on day 0 and taken down day 3. LNA oligonucleotide concentration (µg/g tissue) determined by hybridization-ELISA. Red, LNA 10, random mixture of stereoisomers. Orange, stereo-defined isomers with identical nucleobase sequence and design as LNA 10. Molecules created using Spartan 14,16 (www.wavefun.com).

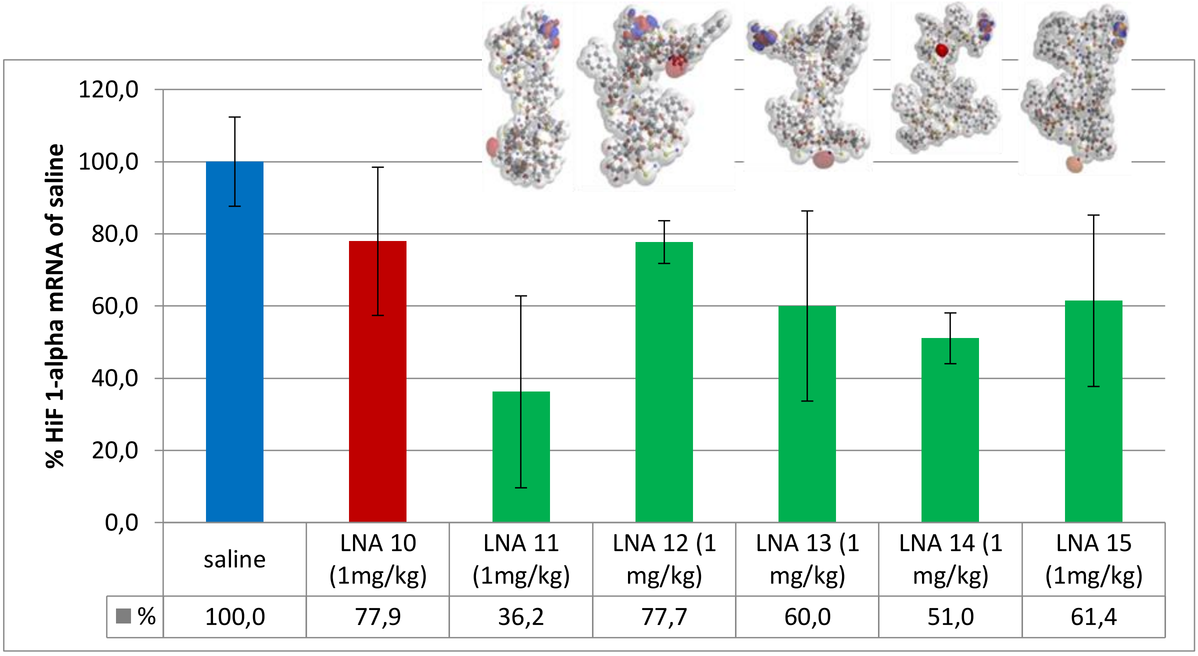

Figure S2. Antisense activity of LNA diastereoisomers targeting Hif-1 alpha. Diversity of diastereoisomer on target downregulation (Hif-1alpha) in livers of mice (C57BL/6qBom) (n=5) dosed once intravenously at 1 mg/kg on day 0 and taken down day 3 (60h). Target mRNA level (% of saline control) were measured in liver tissue. Blue is saline control, red, LNA 10, is the random mixture of stereoisomers. Green is stereo-defined isomers with same nucleobase sequence and design as LNA 10. Molecules created using Spartan 14,16 (www.wavefun.com).

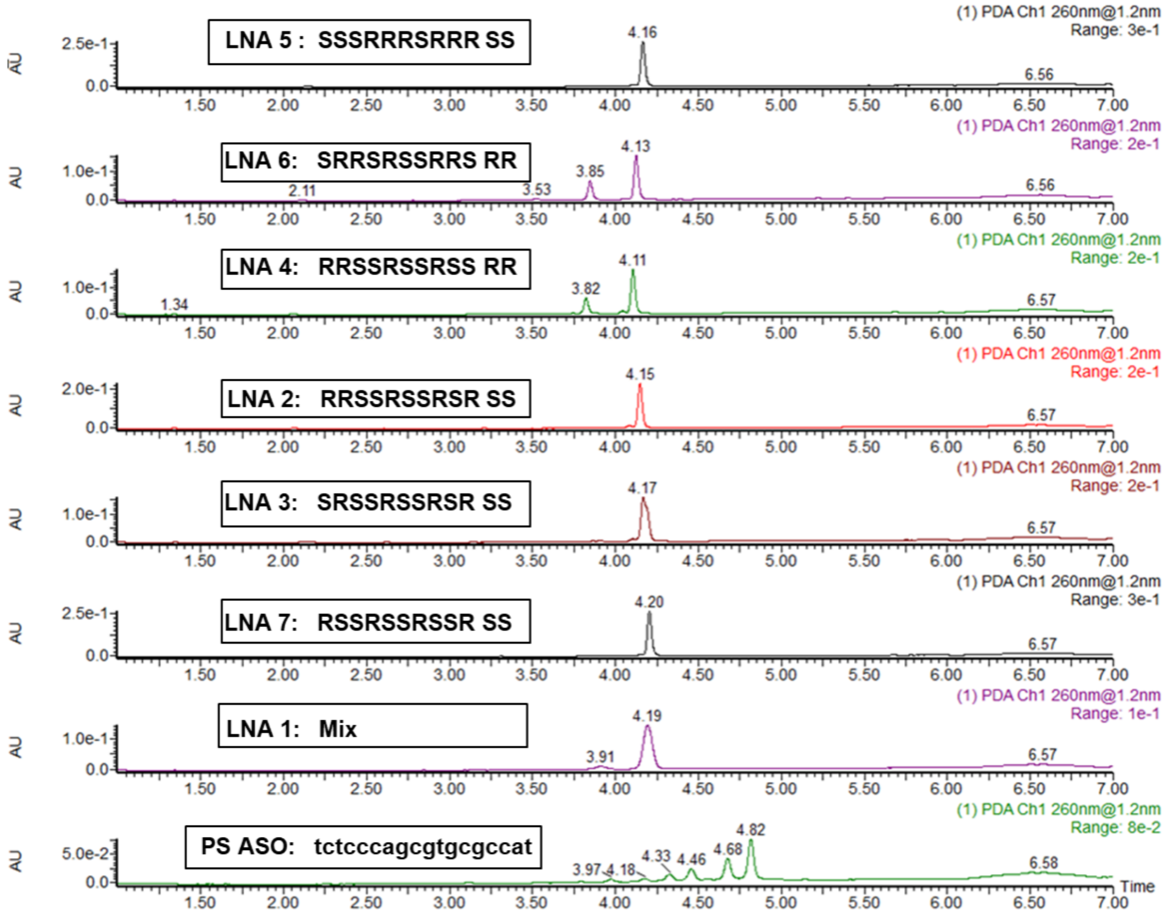

Figure S3. LNA nuclease stability after 4 hours at 37 ºC in rat serum of the stereo-defined versions of the phosphorothioate random mixture LNA 1 (5´-G^{m}CattggtatT^{m}CA-3`). The R configuration of the 3´ phosphorothioate linkages in combination with a 3´-purine LNA are more prone to be cleaved by 3´*exo* nucleases. The degradation products from LNA 4 and LNA 6 are identified as the n-1 (-A) sequences Mz 3968.

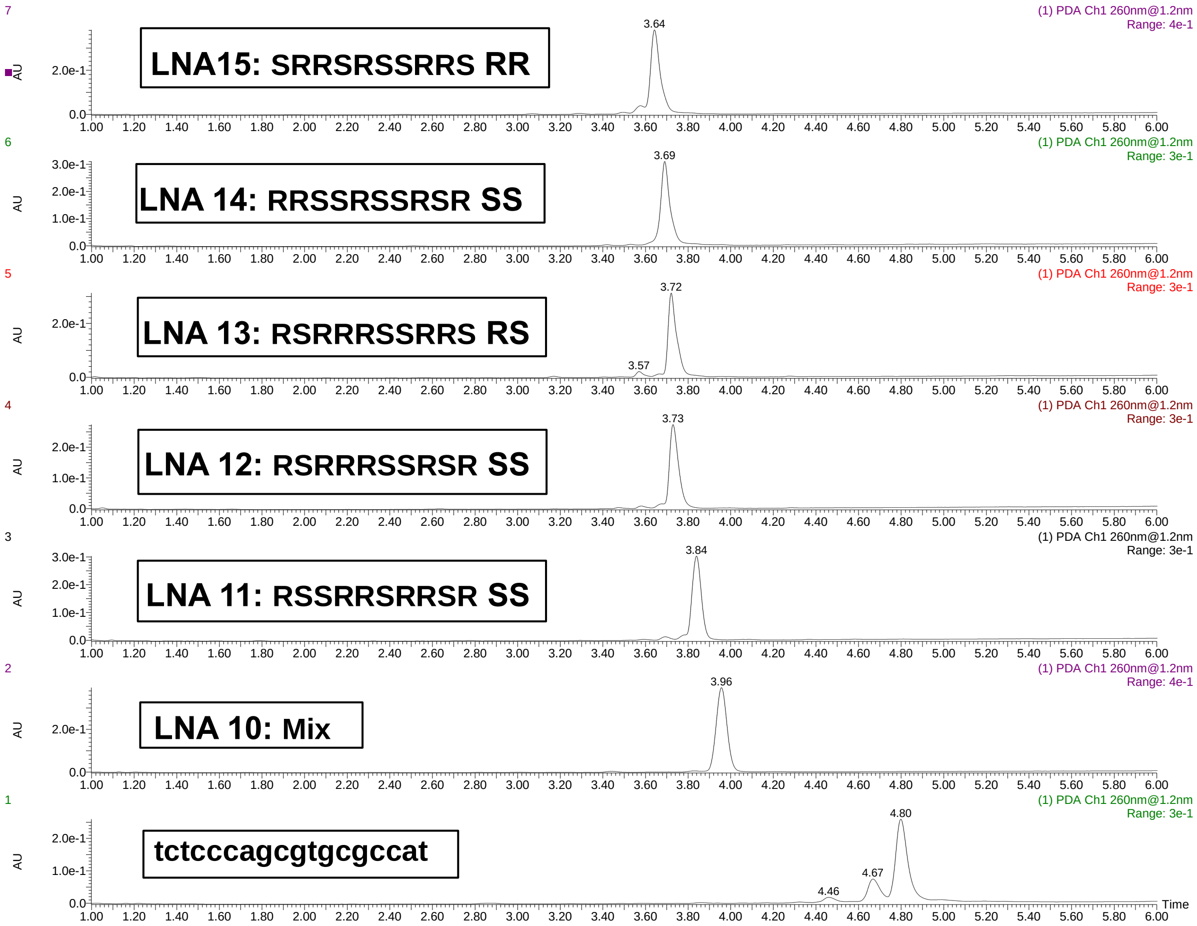

Figure S4. The stability after 4 hours at 37 °C in rat serum of LNA 10 (5´- G^m^CaagcatcctGT-3`) and the stereo-specific versions the phosphorothioate LNA 10-15. The R configuration of the 3´ phosphorothioate are more prone to be cleaved by 3´*exo* nuclease activity but is more stable with the 3`T base than seen above with the 3`A base (Fig. S3).

			Cleaved target (%)
**No.**	**Sequence**	**Chiral sequence**	**Serum (4 h)**
**PS AON**	**tctcccagcgtgcgccat**	**Random mixture**	44.6
**LNA 1**	**G^{m}CattggtatT^{m}CA**	**Random mixture**	5.6
**LNA 2**	**G^{m}CattggtatT^{m}CA**	**RRSSRSSRSR SS**	0
**LNA 3**	**G^{m}CattggtatT^{m}CA**	**SRSSRSSRSR SS**	0
**LNA 4**	**G^{m}CattggtatT^{m}CA**	**RRSSRSSRSS RR**	19.6
**LNA 5**	**G^{m}CattggtatT^{m}CA**	**SSSRRRSRRR SS**	0
**LNA 6**	**G^{m}CattggtatT^{m}CA**	**SRRSRSSRRS RR**	19.2
**LNA 7**	**G^{m}CattggtatT^{m}CA**	**RSSRSSRSSR SS**	2.4
**LNA 10**	**G^{m}CaagcatcctGT**	**Random mixture**	1.3
**LNA 11**	**G^{m}CaagcatcctGT**	**RSSRRSRRSR SS**	0
**LNA 12**	**G^{m}CaagcatcctGT**	**RSRRRSSRSR SS**	0
**LNA 13**	**G^{m}CaagcatcctGT**	**RSRRRSSRRS RS**	0.9
**LNA 14**	**G^{m}CaagcatcctGT**	**RRSSRSSRSR SS**	0
**LNA 15**	**G^{m}CaagcatcctGT**	**SRRSRSSRRS RR**	1.9

Table S1. Relative stabilities of stereo-defined isomers of LNA 1 (random mixture) and LNA 10 (random mixture) after 4 hours in rat serum at 37^{0}.

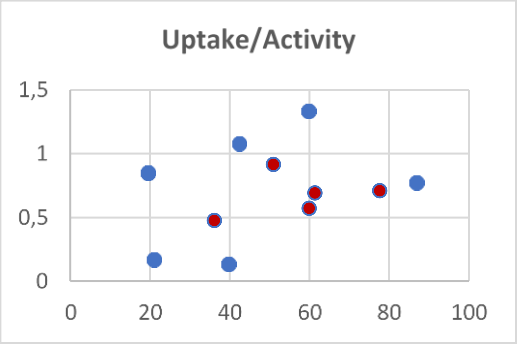
Uptake/Activity
1,5
1
0,5
0
0
20
40
60
80
100

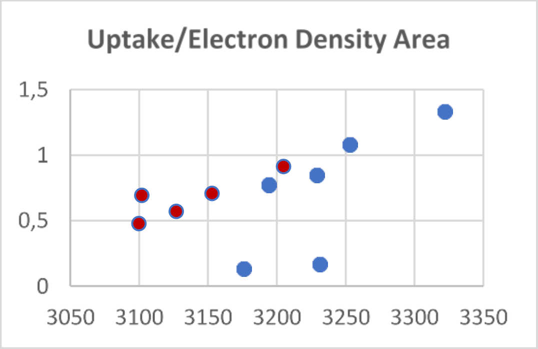
Uptake/Electron Density Area
1,5
1
0,5
0
3050
3100
3150
3200
3250
3300
3350

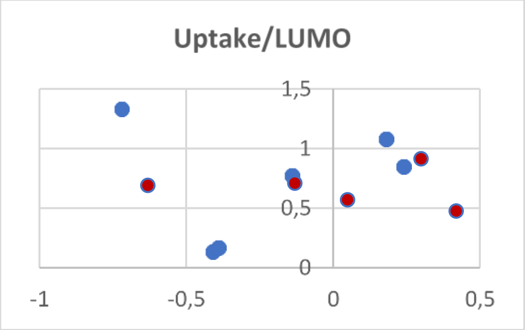
Uptake/LUMO
1,5
1
0,5
0
-1
-0,5
0
0,5

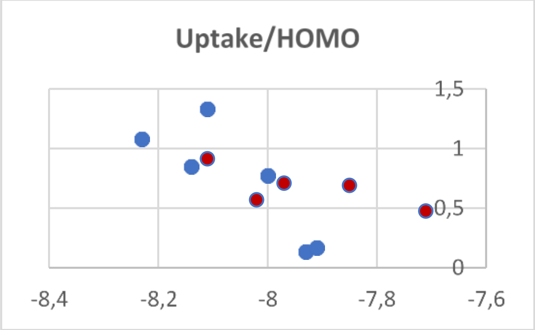
Uptake/HOMO
1,5
1
0,5
0
-8,4
-8,2
-8
-7,8
-7,6

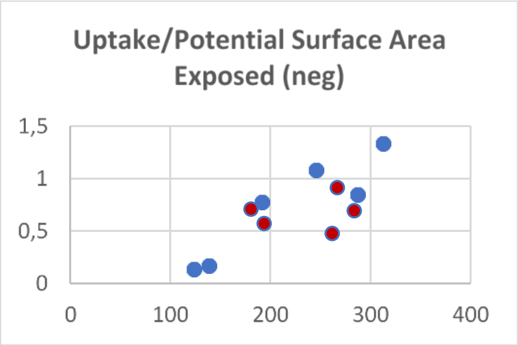
Uptake/Potential Surface Area Exposed (neg)
1,5
1
0,5
0
0
100
200
300
400

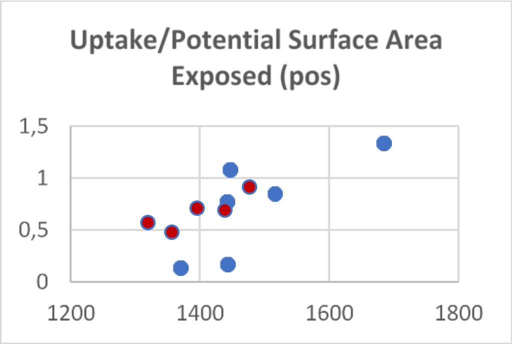
Uptake/Potential Surface Area Exposed (pos)
1,5
1
0,5
0
1200
1400
1600
1800

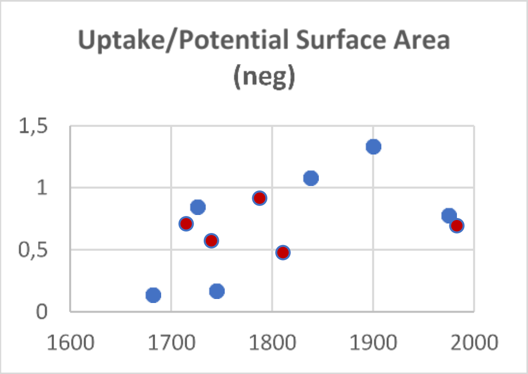
Uptake/Potential Surface Area (neg)
1,5
1
0,5
0
1600
1700
1800
1900
2000

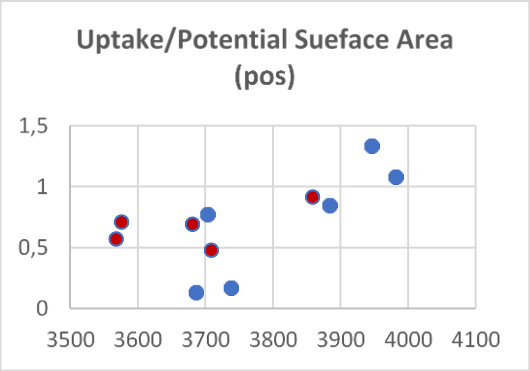
Uptake/Potential Sueface Area (pos)
1,5
1
0,5
0
3500
3600
3700
3800
3900
4000
4100

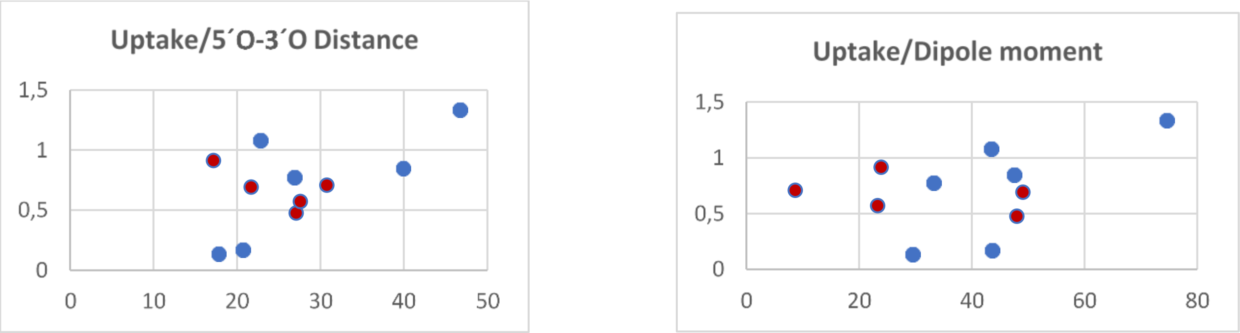

Figure S5. The relations between the calculated structural parameters and liver uptake of the 11 stereospecific LNA oligonucleotides. Read dots are LNAs with the Hif-1-alpha motive and blue dots are LNAs with the ApoB motive.

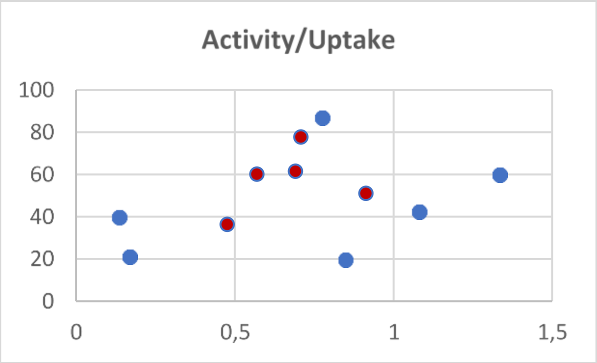
Activity/Uptake
100
80
60
40
20
0
0
0,5
1
1,5

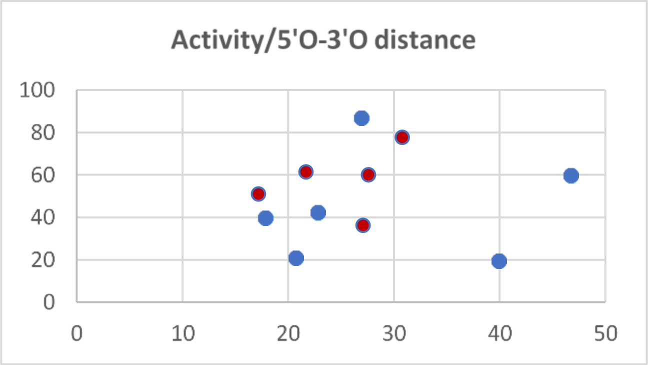
Activity/5'O-3'O distance
100
80
60
40
20
0
0
10
20
30
40
50

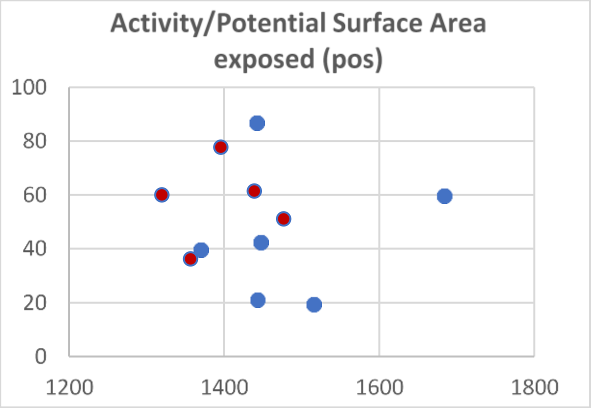
Activity/Potential Surface Area exposed (pos)
100
80
60
40
20
0
1200
1400
1600
1800

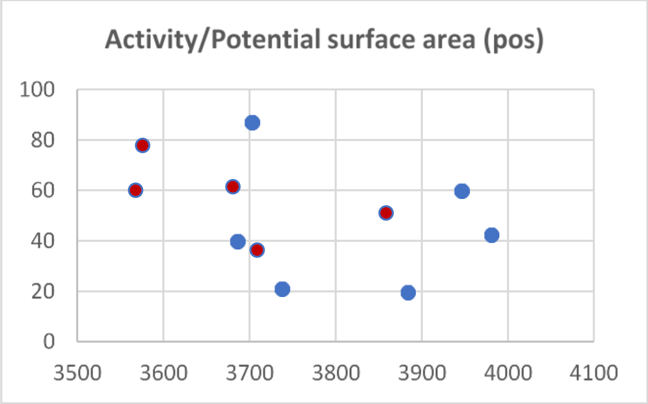
Activity/Potential surface area (pos)
100
80
60
40
20
0
3500
3600
3700
3800
3900
4000
4100

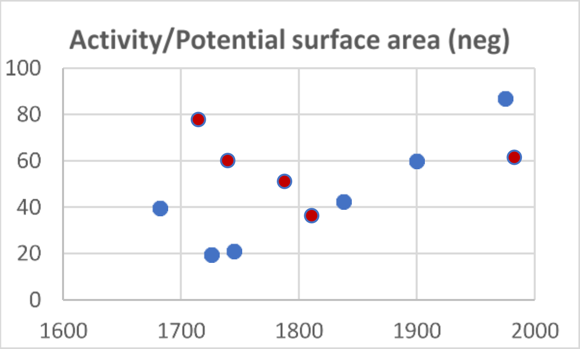
Activity/Potential surface area (neg)
100
80
60
40
20
0
1600
1700
1800
1900
2000

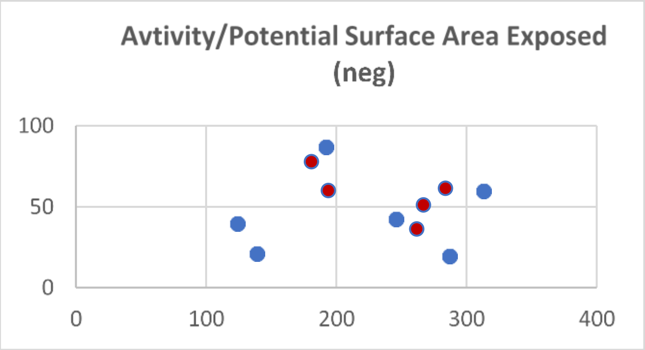
Avtivity/Potential Surface Area Exposed (neg)
100
50
0
0
100
200
300
400

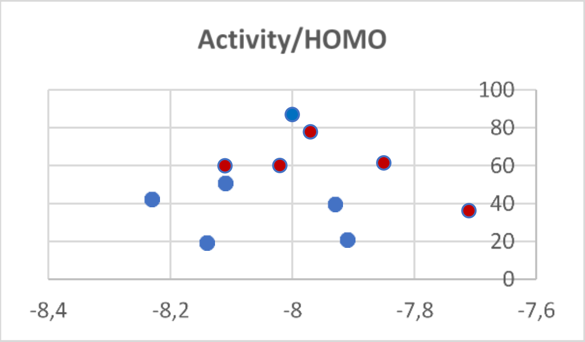
Activity/HOMO
100
80
60
40
20
0
-8,4
-8,2
-8
-7,8
-7,6

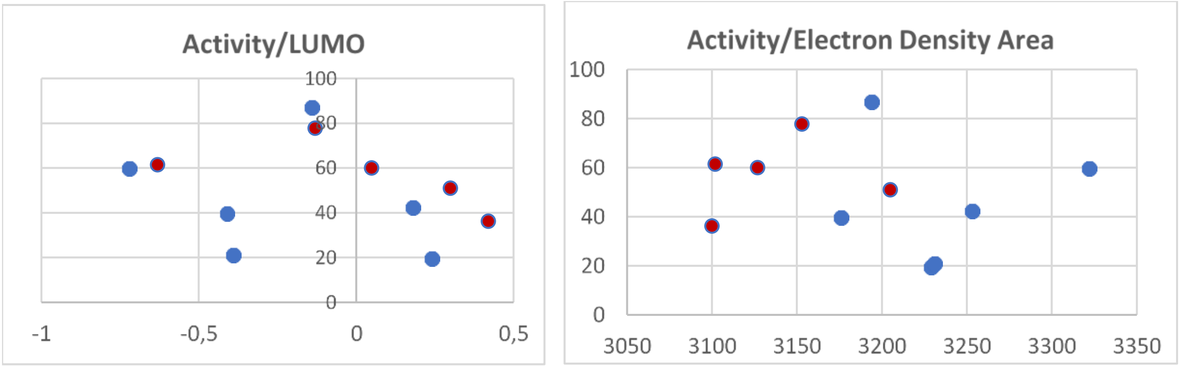

Figure S6. The relations between the mRNA downregulation in the liver and the calculated structure parameters of the 11 stereospecific LNA oligonucleotides. Read dots are LNAs with the Hif-1-alpha motive and blue dots are LNAs with the ApoB motive.
